# Use of a doxycycline-enrofloxacin-metronidazole combination with/without diminazene diaceturate to treat naturally occurring canine babesiosis caused by *Babesia gibsoni*

**DOI:** 10.1186/1751-0147-52-27

**Published:** 2010-04-24

**Authors:** Ming-Yu Lin, Hui-Pi Huang

**Affiliations:** 1 Section of Small Animal Internal Medicine, National Taiwan University Veterinary Hospital, Taipei, Taiwan; 2 Department of Veterinary Medicine, National Taiwan University, National Taiwan University, No. 1, Section 4, Roosevelt Road, Taipei 106, Taiwan

## Abstract

Canine babesiosis is an important worldwide, tick-borne disease caused by hemoprotozoan parasites of the genus *Babesia*. *Babesia gibsoni *is the predominant species that causes canine babesiosis in Taipei, Taiwan. It is a small pleomorphic intraerythrocytic parasite that can cause erythrocyte destruction and hemolytic anemia. Efficacy of oral administration of a doxycycline-enrofloxacin-metronidazole combination with and without injections of diminazene diaceturate in the management of naturally occurring canine babesiosis caused by *B. gibsoni *was evaluated retrospectively. The overall efficacy of this combination of doxycycline-enrofloxacin-metronidazole in conjunction with and without administration of diminazene diaceturate was 85.7% and 83.3%, respectively; with a mean recovery time of 24.2 and 23.5 days, respectively. Concomitant use of intramuscular diminazene diaceturate may not improve the efficacy of a doxycycline-enrofloxacin-metronidazole combination in management of canine babesiosis caused by *B. gibsoni*.

## Findings

Canine babesiosis is an important worldwide, tick-borne disease caused by hemoprotozoan parasites of the genus *Babesia*, and the predominant species infecting dogs is *B. vogeli *[1]. In a previous study, *B. gibsoni *was found to be the major causative agent of canine babesiosis in Taipei, Taiwan [2]. *Babesia gibsoni *is a small pleomorphic, intraerythrocytic parasite that can cause erythrocyte destruction and hemolytic anemia [1]. This parasite is suspected to be transmitted through the bite of ixodid ticks, such as *Rhipicephalus sanguineus *[1,3-8].

Clinical signs of canine babesiosis are characterized by lethargy, anorexia, fever, hemolytic anemia, thrombocytopenia, splenomegaly and even septic shock [2,9-14]. Chronic infection is common. Definitive diagnosis of canine babesiosis is based on medical history and clinical signs together with the identification of *Babesia *spp. within infected erythrocytes, positive serologic results, and detection of amplification of nucleic acid extracted from blood or tissues [4,5,15].

Many drugs have been applied in management of canine babesiosis, including babesiacidal agents (diminazene aceturate, imidocarb diproprionate), antibiotics (azithromycin, clindamycin, doxycycline, metronidazole), and an antiprotozoal agent (Atovaquone). However, no single drug has successfully eliminated *B. gibsoni *from infected dogs [9,10,12,13,15-18]. Addictive or synergistic effects of these drugs in combination have not been fully evaluated. The babesiacide diminazene diaceturate is mainly used in horses, donkeys and cows [19-21]. Efficacy of diminazene diaceturate in management of canine babesiosis is limited, but this is the only diminazene available for veterinary use in Taiwan, whereas atovaquone is not yet available. The aim of this study was to evaluate the efficacy of a doxycycline-enrofloxacin-metronidazole (D-E-M) combination with and without administration of diminazene diaceturate to manage naturally occurring canine babesiosis caused by *B. gibsoni*.

Medical records of dogs in which babesiosis was diagnosed at the National Taiwan University Veterinary Hospital from January 2005 to December 2006 were reviewed. Inclusion criteria were history of tick infestation, presence of anemia, and identification of *B. gibsoni *in the stained blood films, and treatment with an oral doxycycline (7-10 mg/Kg, q 12 hours; China Chemical & Pharmaceutical Co. Ltd., Taipei, Taiwan)-enrofloxacin (2-2.5 mg/Kg, q 12 hours; Baytril^®^, Bayer HealthCare AG, Leverkusen, Germany)-metronidazole (5-15 mg/Kg, q 12 hours; Sinphar Pharmaceutical Co. Ltd. Taipei, Taiwan; D-E-M) combination of six weeks with and without two intramuscular injections of diminazene diaceturate (3 mg/Kg, q 7 days; Tricip Plus^®^, Cipla Ltd., Mumbai, India). Informed consent of using diminazene diaceturate had been obtained from the owners of dogs. Breed, sex, age, pre-treatment complete cell counts, bi-weekly follow-up complete cell counts, and the outcome for each case were recorded. Co-infection with other tick-borne diseases, such as *Anaplasma phagocytophilum *and *Ehrlichia canis *were excluded using a commercially available immunoenzymatic dot-ELISA assays (Snap 4Dx, Idexx Laboratories Inc., Maine, U.S.A.).

In this study, identification of *B. gibsoni *was based on morphological characteristics of intraerythrocytic parasites found in the stained blood films. Dogs affected with other babesia species, such as *B. canis *and *B. vogeli *were excluded from this study. Efficacy of treatment was defined as elimination of *B. gibsoni *in blood films, and clinical remission for seven weeks after the treatment was discontinued.

Comparison of the time to reach clinical remission between two treatment protocols was performed by means of repeated-measures ANOVAs. Associations between sex, age, treatment protocols, and time to reach clinical remission were evaluated by means of ANOVA. Variables that might have influenced relapse of anemia and persistent parasitemia were analyzed by use of a Cox proportional hazard model. Such variables included sex, age and severity of anemia at diagnosis, and treatment protocol. Data for dogs that failed to response (relapse of anemia and persistent parasitemia) were censored. All statistical analyses were performed using SPSS software (Statistical Package for the Social Sciences, version 13.0; SSPS Inc, Illinois, USA). Continuous data are presented as mean ± SD. Statistical significance was set at *P *≤ 0.05.

Medical records of 6789 dogs from January 2005 to December 2006 were screened. Of these, 118 cases was diagnosed as babesiosis and caused by *B. gibsoni*. Breeds of the 40 dogs included in this study were: 10 Maltese terriers; four each of Beagles, mixed breeds and miniature Schnauzers; three of Labrador retrievers and Yorkshire terriers; two each of Pit bull terriers, Golden retrievers, and Pomeranians; one each of Chihuahua, Siberian Husky, Old English sheepdog, miniature Poodle, Pug and Shih Tzu. The mean age of the dogs included in the study was 5.3 ± 3.9 years, and ranged from 9 months to 14 years old. The ratio of male to female was 3 to 1.

Twenty-eight dogs were treated with an oral combination of D-E-M for six weeks plus two intramuscular injections of diminazene diaceturate, whereas 12 dogs received only the oral combination of D-E-M for six weeks.

Of 28 dogs (mean age: 6.0 ± 3.8 years) treated with oral D-E-M combination and two intramuscular injections of diminazene diaceturate, 4 dogs showed relapse of anemia and parasitemia after 14, 28, 35 and 42 days, respectively, and 24 dogs had clinical remission. The overall efficacy of multiple antibiotics combined with diminazene diaceturate was 85.7%, with a mean recovery time of 24.2 days (n = 24, Table 1).

**Table 1:** Hematological parameters (mean and standard deviation, SD) in 24 dogs showing clinical remission for babesiosis following treatment with a doxycycline-enrofloxacin-metronidazole combination and intramuscular diminazene diaceturate.

Parameter	Reference Range	Day 0 Mean (SD)	Day 14 Mean (SD)	Day 28 Mean (SD)	Day 90 Mean (SD)
RBC (×10^6^/μL)	5.2-7.9	2.7 (1.7)	3.7 (1.6)	5.3 (1.1)	5.8 (0.5)
Hb (g/dL)	11.6-17.1	6.8 (4.7)	8.2 (3.8)	11.5 (1.5)	12.2 (0.8)
Hct (%)	38.4-53.1	21.5 (10.3)	29.3 (7.4)	34.7 (6.9)	38.2 (4.8)

RBC, red blood cell count; Hb haemoglobin; Hct: haematocrit.

Of 12 dogs (mean age: 3.6 ± 3.8 years) treated with D-E-M, but were not given injections of diminazene diaceturate relapsed anemia was identified in 2 dogs after 45 and 73 days, while another 10 dogs reached clinical remission. The overall efficacy of management of canine babesiosis using only multiple antibiotics was 83.3%, with a mean recovery time of 23.5 days (n = 10, Table 2).

**Table 2:** Hematological parameters (mean and standard deviation, SD) in 10 dogs showing clinical remission for babesiosis following treatment with a doxycycline-enrofloxacin-metronidazole combination.

Parameter	Reference Range	Day 0 Mean (SD)	Day 14 Mean (SD)	Day 28 Mean (SD)	Day 90 Mean (SD)
RBC (10^6^/μL)	5.2-7.9	2.8 (1.9)	3.8 (1.7)	5.1 (1.4)	5.6 (0.5)
Hb (g/dL)	11.6-17.1	6.8 (5.1)	8.3 (4.3)	11.3 (1.6)	12.1 (0.9)
Hct (%)	38.4-53.1	21.9 (10.8)	29.2 (6.8)	33.8 (7.2)	39.6 (5.6)

RBC, red blood cell count; Hb haemoglobin; Hct: haematocrit.

No significant difference was seen in management efficacy between the two protocols (*P *= 0.36). The time to reach clinical remission was not affected by sex, age or treatment protocols (*P *= 0.88, *P *= 0.36, *P *= 0.38, respectively).

Results of the Cox proportional hazard model indicated that relapse of anemia and persistent parasitemia was not associated with sex, age or severity of anemia at diagnosis, or treatment protocol (*P *= 0.72, *P *= 0.72, *P *= 0.65, *P *= 0.52, respectively)

In this study, the overall prevalence of canine babesiosis caused by *B. gibsoni *was 1.7%, which was only slightly higher than the prevalence established in a similar study conducted 10 years earlier (1.6%) [2].

In our previous study, Maltese terriers seemed to be predisposed to babesiosis [2]. In the present study, the Maltese terrier was accounting for 25% of the cases of babesiosis, but no further statistical analyses were carried out to assert that Maltese terrier was predisposing to canine babesiosis. Maltese terrier is, however, much less popular compared to 10 years ago. A large scale study of breed predisposition to this disease is warranted. Certain breeds, such as American Pit Bull terriers and Tosa dogs [5,22], have also been found to having higher rates of subclinical infection with *B. gibsoni*. Particularly dogs that have been bitten were found to have a higher subclinical infection with *B. gibsoni *[5,22,23].

The efficacy of treatment of babesiosis caused by *B. gibsoni *with either doxycycline or metronidazole has been evaluated, but the ability of each drug alone to successfully treat *B. gibsoni *is still controversial [18]. In the present study, synergistic efficacy of the D-E-M combination was 83.3%. Relapse of anemia and persistent parasitemia was not associated with sex, age or severity of anemia at diagnosis, or D-E-M combination with or without diminazene diaceturate.

Diminazene aceturate, an aromatic diamidine compound, can interfere with the synthesis of DNA and aerobic glycolysis and has been commonly used against infection of canine babesiosis caused by *B. gibsoni *[18]. Diminazene diaceturate has also been widely used against infection of babesiosis in horses, donkeys and cows [13,20,21]. In a comparative study, diminazene aceturate exhibited a higher efficacy than diminazene diaceturate in the treatment of babesiosis in horses [21]. In the present study, efficacy and the time to achieve clinical remission did not differ between the two treatment protocols. Although the efficacy of the administration of diminazene diaceturate alone against *B. gibsoni *was not evaluated in our study, it suggested that concomitant use of diminazene diaceturate may not improve the efficacy of a D-E-M combination treatment of canine babesiosis caused by *B. gibsoni*.

All cases of subclinical infection of *B. gibsoni *may not be detected solely by, morphological identification in blood smears, and polymerase chain reaction assay should therefore be included in further studies in order to identify subclinical carriers after treatment.

Concomitant use of intramuscular diminazene diaceturate may not improve the efficacy of a doxycycline-enrofloxacin-metronidazole combination in management of canine babesiosis caused by *B. gibsoni*.

## Competing interests

The authors declare that they have no competing interests.

## Authors' contributions

MYL participated in the designs of the study and carried out the recruitment of cases. She also drafted the manuscript. HPH participated in the designs of the study, carried out the recruitment of cases and performed the statistical analysis and the manuscript writing. Both authors read and approved the final manuscript.

## References

[B1] Uilenberg G *Babesia *- a historical overview Vet Parasitol 2006 138 3 10 10.1016/j.vetpar.2006.01.035 16513280

[B2] Chen YP Huang HP Prevalence and laboratory findings of canine tick-borne diseases in the National Taiwan University Veterinary Hospital Taiwan Vet J 2003 29 97 103

[B3] Rajamanickam C Wiesenhutter E Zin FMD Hamid J The incidence of canine haematozoa in Peninsular Malaysia Vet Parasitol 1985 17 151 157 10.1016/0304-4017(85)90101-3 3922103

[B4] Inokuma H Yoshizaki Y Matsumoto K Okuda M Onishi T Nakagome K Kosugi R Hirakawa Molecular survey of *Babesia *infection in dogs in Okinawa, Japan Vet Parasitol 2004 121 31 346 10.1016/j.vetpar.2004.03.012 15135876

[B5] Matsuu A Kawabe A Koshida Y Ikadai H Okano S Higuchi S Incidence of canine *Babesia gibsoni *infection and subclinical infection among Tosa dogs in Aomori Prefecture, Japan J Vet Med Sci 2004 66 893 897 10.1292/jvms.66.893 15353837

[B6] Miyama T Sakata Y Shimada Y Ogino S Watanabe M Itamoto K Okuda M Verdida RA Xuan X Nagasawa H Inokuma H Epidemiological survey of *Babesia gibsoni *infection in dogs in eastern Japan J Vet Med Sci 2005 67 467 471 10.1292/jvms.67.467 15942130

[B7] Dantas-Torres F Figueredo LA Canine babesiosis: a Brazilian perspective Vet Parasitol 2006 141 197 203 10.1016/j.vetpar.2006.07.030 16962707

[B8] Trapp SM Messick JB Vidotto O Jojima FS de Morais HAS *Babesia gibsoni *genotype Asia in dogs from Brazil Vet Parasitol 2006 141 177 180 10.1016/j.vetpar.2006.04.036 16765518

[B9] Vercammen F De Deken R Maes L Prophylactic treatment of experimental canine babesiosis (*Babesia canis*) with doxycycline Vet Parasitol 1996 66 251 255 10.1016/S0304-4017(96)01016-3 9017887

[B10] Wulansari R Wijaya A Ano H Horii Y Nasu T Yamane S Makimura S Clindamycin in the treatment of *Babesia gibsoni *infections in dogs J Am Anim Hosp Assoc 2003 39 558 562 14736722 10.5326/0390558

[B11] Gopegui RR Penalba B Goicoa A Espada Y Fidalgo LE Espino L Clinico-pathological findings and coagulation disorders in 45 cases of canine babesiosis in Spain Vet J 2006 174 129 132 16901737 10.1016/j.tvjl.2006.05.017

[B12] Vial HJ Gorenflot A Chemotherapy against babesiosis Vet Parasitol 2006 138 147 160 10.1016/j.vetpar.2006.01.048 16504402

[B13] Suzuki K Wakabayashi H Takahashi M Fukushima M Yabuki A Endo Y A possible treatment strategy and clinical factors to estimate the treatment response in *Babesia gibsoni *infection J Vet Med Sci 2007 69 563 568 10.1292/jvms.69.563 17551236

[B14] Matijatko V Kiš I Torti M Brkljačić M Kučer N Rafaj RB Grden D Živičnjak T Mrljak V Septic shock in canine babesiosis Vet Parasitol 2009 162 263 270 10.1016/j.vetpar.2009.03.011 19345507

[B15] Sakuma M Setoguchi A Endo Y Possible emergence of drug-resistant variants of *Babesia gibsoni *in clinical cases treated with atovaquone and azithromycin J Vet Intern Med 2009 23 493 498 10.1111/j.1939-1676.2009.0300.x 19645835

[B16] Birkenheuer AJ Levy MG Breitschwerdt EB Efficacy of combined atovaquone and azithromycin for therapy of chronic *Babesia gibsoni *(Asian genotype) infections in dogs J Vet Intern Med 2004 18 494 498 15320586 10.1892/0891-6640(2004)18<494:eocaaa>2.0.co;2

[B17] Matsuu A Koshida Y Kawahara M Inoue K Ikadai H Hikasa Y Okano S Higuchi S Efficacy of atovaquone against *Babesia gibsoni *in vivo and in vitro Vet Parasitol 2004 124 9 18 10.1016/j.vetpar.2004.07.005 15350657

[B18] Matsuu A Yamasaki M Xuan X Ikadai H Hikasa Y In vitro evaluation of the growth inhibitory activities of 15 drugs against *Babesia gibsoni *(Aomori strain) Vet Parasitol 2008 157 1 8 10.1016/j.vetpar.2008.07.023 18771856

[B19] Singh B Banerjee DP Gautam OP Comparative efficacy of diminazene diaceturate and imidocarb dipropionate against *Babesia equi *infection in donkeys Vet Parasitol 1098 7 173 179 10.1016/0304-4017(80)90020-5

[B20] Turnipseed SB Clark SB Andersen WC Karbiwnyk CM Miller KE Hurlbut JA Confirmation of diminazene diaceturate in bovine plasma using electrospray liquid chromatography-mass spectrometry J Chromatogr B 2006 844 127 133 10.1016/j.jchromb.2006.07.016 16891161

[B21] Rashid HB Chaudhry M Rashid H Pervez K Khan MA Mahmood AK Comparative efficacy of diminazene diaceturate and diminazene aceturate for the treatment of babesiosis in horses Trop Anim Hlth Prod 2008 40 463 467 10.1007/s11250-007-9121-2 18575975

[B22] Birkenheuer AJ Correa MT Levy MG Breitschwerdt EB Geographic distribution of babesiosis among dogs in the United States and association with dog bites: 150 cases (2000-2003) J Am Vet Med Assoc 2005 227 942 947 10.2460/javma.2005.227.942 16190594

[B23] Macintire DK Boudreaux MK West GD Bourne C Wright JC Conrad PA *Babesia gibsoni *infection among dogs in the southeastern United States J Am Vet Med Assoc 2002 220 325 329 10.2460/javma.2002.220.325 11829262

